# Characteristics Of Hikikomori Cases Aged 50-64 Receiving Financial And Daily Support From Older Parents In Japan

**DOI:** 10.1093/geroni/igaf122.2487

**Published:** 2025-12-31

**Authors:** Kiyoko Fukushima, Noriko Tsukada, Asako Katsumata

**Affiliations:** Japan Lutheran College, Mitaka, Tokyo, Japan; Nihon University, Tokyo, Tokyo, Japan; Japan Lutheran College, Mitaka, Tokyo, Japan

## Abstract

This study examines the characteristics of hikikomori cases aged 50-64 in Japan by comparing them with cases aged 15-49. Japan has approximately 613,000 socially withdrawn individuals aged 40-64, with 91,000 never leaving home. Using data from a February 2024 survey of 1,735 municipal social welfare councils (response rate: 28.4%), we analyzed 312 cases where support led to observable changes. Among them, 81 cases (26.0%) involved individuals aged 50-64. Findings show that 78.8% were male, 61.5% had work experience, and 82.3% had an illness or disability. Before support, 85.6% lived with their family, and after support, 72.7% lived with their family. Even after support, 35.8% lived with their father and 55.1% lived with their mother. Compared to the 15-49 age group, those aged 50-64 were significantly more likely to have work experience (p < 0.001), less likely to have experienced truancy (p < 0.001), and less likely to live with family (p < 0.001). The duration of social withdrawal was significantly longer in the 50-64 group (17.3 years vs. 10.6 years, p < 0.001). However, no significant differences were observed in gender, education, illness, living arrangements, or family acceptance levels (e.g., their perception of hikikomori status). Despite longer withdrawal periods, post-support recovery time showed no significant differences between age groups. These results highlight the importance of early intervention through community outreach to foster change. Strengthening trust-based relationships and multi-organizational collaboration is crucial for supporting aging parents and their withdrawn adult children in reconnecting with society.

